# Prevalence of Gender-Affirming Surgical Procedures Among Minors and Adults in the US

**DOI:** 10.1001/jamanetworkopen.2024.18814

**Published:** 2024-06-27

**Authors:** Dannie Dai, Brittany M. Charlton, Elizabeth R. Boskey, Landon D. Hughes, Jaclyn M.W. Hughto, E. John Orav, Jose F. Figueroa

**Affiliations:** 1 Department of Health Policy and Management, Harvard T.H. Chan School of Public Health, Boston, Massachusetts; 2 Department of Population Medicine, Harvard Medical School and Harvard Pilgrim Health Care Institute, Boston, Massachusetts; 3 Division of Gynecology, Boston Children's Hospital, Boston, Massachusetts; 4 Departments of Behavioral and Social Sciences and Epidemiology, Harvard T.H. Chan School of Public Health, Boston, Massachusetts; 5 Department of Behavioral and Social Sciences and Epidemiology, Brown School of Public Health, Providence, Rhode Island; 6 Department of Biostatistics, Harvard T. H. Chan School of Public Health, Boston, Massachusetts

## Abstract

This cross-sectional study uses a national dataset to examine the prevalence of gender-affirming surgical procedures among minors and adults in the US.

## Introduction

Gender-affirming health care aims to align an individual’s physical gender expression with their intrinsic gender identity. An example is breast reduction for cisgender males with gynecomastia. Recently, there have been increased legislative efforts restricting gender-affirming health care specifically for transgender and gender diverse (TGD) people.^1^ Proponents of these efforts express concerns that TGD minors may be too commonly using gender-affirming surgical procedures. Given stringent clinical standards for gender-affirming care,^2,3^ use of gender-affirming surgical procedures by TGD minors is expected to be low. However, there are limited national data; while 1 study provided national estimates, it did not capture minors under the age of 12 years and did not exclude procedures with clinical justifications outside of gender affirmation.^4^ Therefore, using a national dataset, we evaluated the extent to which TGD minors and adults received gender-affirming surgical procedures in 2019. Additionally, in line with a recent publication from bioethicists to acknowledge that gender-affirming care is used by people of all genders,^5^ we provide a novel characterization of the relative use of 1 gender-affirming surgery—breast reductions—by TGD and cisgender populations.

## Methods

This cross-sectional study was approved by the institutional review board at the Harvard T.H. Chan School of Public Health. This study followed the Strengthening the Reporting of Observational Studies in Epidemiology (STROBE) reporting guideline. Informed consent was waived because data were deidentified. Using medical claims from Inovalon Insights, we identified TGD people who received a gender-affirming procedure using previously validated sets of diagnostic and procedural codes in 2019.^6^ We then calculated the rate of people who received a gender-affirming procedure with a TGD-related diagnosis per 100 000 total people in the following categories: adults (18 years or older) or minors (15 to 17 years, 13 to 14 years, and 12 years or younger). Next, given that breast reduction for male cisgender adults and minors can be considered gender-affirming care and can be covered by insurance, we compared the proportion of breast reductions used by cisgender males—males without a TGD-related diagnosis— and TGD people. Importantly, all surgical procedures among patients with indications of differences in sex development or patients with other medical indications for surgery (eg, cancer, injury) were excluded (eMethods and eTable in Supplement 1). Analyses were performed with Microsoft SQL 2019 (Microsoft). Data analysis was conducted from June 2023 to January 2024.

## Results

**Figure 1.**
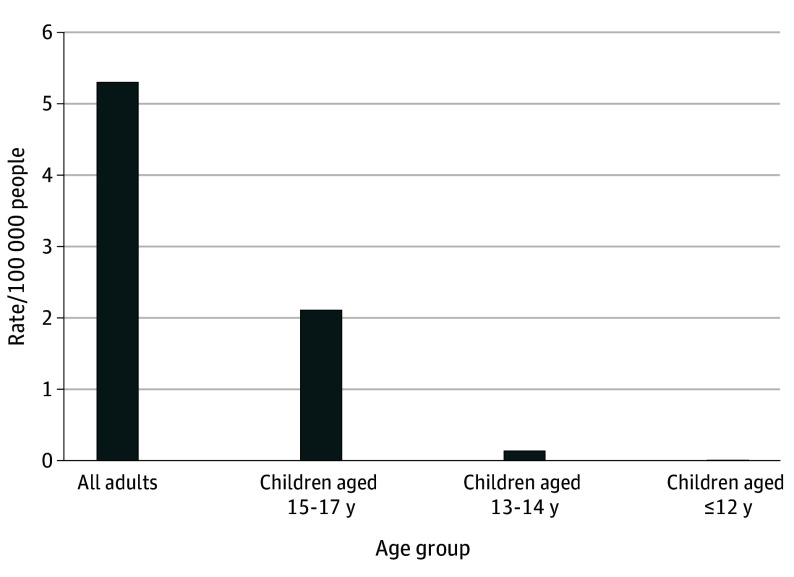
Rates of Gender-Affirming Surgical Procedures Associated With a Transgender and Gender Diverse (TGD)-Related Diagnosis per 100 000 People in 2019 Each bar represents the number of people in each age group who received a gender-affirming surgery associated with a TGD-related diagnosis, per 100 000 total people in that age group in 2019.

**Figure 2.**
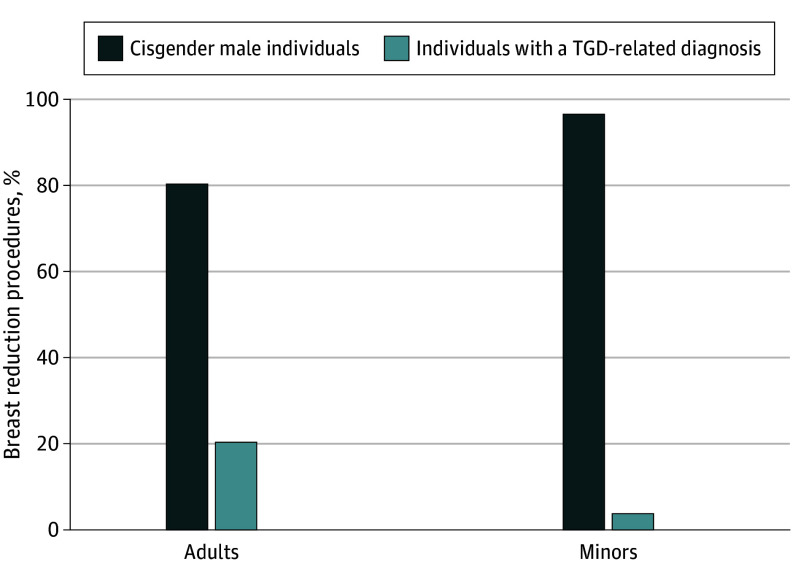
Proportion of Breast Reductions Performed on Males Without a Transgender and Gender Diverse (TGD)-Related Diagnosis and TGD People, 2019 Each bar represents the proportional use of gender-affirming breast reductions by people with a TGD-related diagnosis and cisgender males without a TGD-related diagnosis. Breast reductions conducted on those with a TGD-related diagnosis within 6 months of the surgery are assumed to have occurred in a TGD population, and those without are assumed to have occurred in a cisgender population.

In 2019, the sample included 47 437 919 adults who were insured and 22 827 194 minors who were insured, of which 3 835 726 minors (16.8%) were aged 15 to 17 years, 2 708 166 (11.9%) were aged 13 to 14 years, and 16 283 302 (71.3%) were aged 12 years or younger. The rate of undergoing a gender-affirming surgery with a TGD-related diagnosis was 5.3 per 100 000 total adults compared with 2.1 per 100 000 minors aged 15 to 17 years, 0.1 per 100 000 minors aged 13 to 14 years, and 0 procedures among minors aged 12 years or younger (Figure 1). Of gender-affirming surgical procedures identified among adults and minors, 1591 of 2664 (59.7%) and 82 of 85 (96.4%) were chest-related procedures, respectively. Of the 636 breast reductions among cisgender male and TGD adults, 507 (80%) were performed on cisgender males. Of the 151 breast reductions among cisgender male minors and TGD minors, 146 (97%) were performed on cisgender male minors (Figure 2).

## Discussion

In this cross-sectional study of a national insured population in 2019, there were no gender-affirming procedures conducted on TGD minors aged 12 years and younger, and procedures on TGD minors older than 12 were rare and almost entirely chest-related procedures. Additionally, when considering breast reductions among cisgender males and TGD people—a surgery that can be considered gender-affirming among both populations—most were performed on cisgender males. Thus, these findings suggest that concerns around high rates of gender-affirming surgery use, specifically among TGD minors, may be unwarranted. Low use by TGD people likely reflects adherence to stringent standards of gender-affirming care.^2,3^

This study is limited by the reliance on diagnostic and procedure codes in claims data to assume clinical justifications for surgery and the TGD or cisgender identity of patients. These methods may lead to potential misclassifications. Additionally, our findings are limited to insurance-covered use and not self-paid use.

## Notes

Supplement 1.
eMethods.
**eTable. **Gender Affirming Procedure Codes by Procedure Type

Supplement 2.
Data Sharing Statement
