# Supplementary material for: Prevalence of Gender-Affirming Surgical Procedures Among Minors and Adults in the US
Source: JAMA Netw Open. 2024 Jun 27;7(6):e2418814. doi: 10.1001/jamanetworkopen.2024.18814 (PMC11211955; doi:10.1001/jamanetworkopen.2024.18814)
Supplement: Supplement 1. — eMethods. eTable. Gender Affirming Procedure Codes by Procedure Type [file jamanetwopen-e2418814-s001.pdf]

Supplemental Online Content

Dai D, Charlton BM, Boskey ER, et al. Prevalence of gender-affirming surgeries among minors and adults in the US. *JAMA Netw Open*. 2024;7(6):e2418814.
doi:10.1001/jamanetworkopen.2024.18814

eMethods.

eTable. Results of quality assessment per study

This supplemental material has been provided by the authors to give readers additional information about their work.

© 2024 Dai D, et al. *JAMA Network Open.*

eMethods.

Data:

This study used 2019 data from Inovalon Insight's Real-World Data (RWD), which captures administrative insurance claims of people in Medicaid managed care organizations, Medicare Advantage, and commercial insurance across all 50 states. Patients' race and ethnicity was missing for the majority of people and was not included in this study. The study sample was limited to people with active insurance enrollment in 2019 who had at least one insurance-covered medical claim within the year.

Identifying gender-affirming procedures among Transgender and Gender Diverse (TGD) people

Gender-affirming procedures were defined using lists of procedures—using International Classification of Diseases Tenth Revision (ICD-10) procedure codes or Current Procedural Terminology (CPT) codes—from multiple sources including Blue Cross Blue Shield's "Gender Affirming Services", the Centers of Medicare and Medicaid Services "Gender Reassignment Services for Gender Dysphoria", Aetna's "Gender Affirming Surgery", and a previously validated list from Hughto and colleagues.[1-4] (eTable1). To identify TGD people, we used a list of TGD-related ICD-10 diagnoses codes from prior studies and insurance coverage specifications.[1-3,5,6] (eTable2)

Of note, clinical guidelines and insurance companies generally require that TGD people have a TGD-related diagnosis prior to initiating gender-affirming procedures.[7] Thus, we identified gender-affirming procedures on TGD people as a gender-affirming surgery on a patient who had TGD-related diagnosis within 6 months of the surgery. We then retained only surgeries where patients had no other medical indications which could clinically justify the type of procedure done within 6 months of the surgery (exclusionary diagnoses for each procedure-type can found below in eTable 3). eTable 4 contains a list of diagnoses which, if present within 6 months of a patient's surgery, resulted in manual review of patient history by clinical experts to determine if the surgery could definitively be deemed gender-affirming. Lastly, we excluded all surgeries done on intersex individuals to align with proposed legislative regulations which explicitly do not restrict procedures on intersex minors.[8] (eTable 5) It should be noted that these legislative allowances for surgeries on intersex minors stands contrary to growing international human rights agreements which prohibit non-consensual medical procedures on young intersex people.[9] After these steps, we calculate the annual rate at which people underwent gender-affirming surgeries with a TGD-related diagnosis per 100,000 overall people within specific age categories. Additionally, we report the proportion of gender-affirming surgeries among TGD people that were chest-related procedures.

We also determined the number of breast reductions which occurred annually across TGD people and cisgender males. We first identified all breast reduction surgeries (CPT codes 19318, 19300). If the patient had a TGD-related diagnosis within 6 months of the surgery, they were considered TGD; those without a TGD-related diagnosis within 6 months of the surgery and a male sex marker were considered cisgender males. Again, we excluded intersex individuals, as well as any patients with other medical indications for a chest-related procedure within 6 months

© 2024 Dai D, et al. *JAMA Network Open.*

of the surgery (see "Chest" procedure-type exclusionary diagnoses in eTable 3). We then determined the proportion of breast reductions performed on cisgender males and TGD people.

© 2024 Dai D, et al. *JAMA Network Open.*

eTable. Gender Affirming Procedure Codes by Procedure Type

Procedure Type	Code Type	Code	Description
No Exclusionary Dx	ICD-10	0W4M070	Creation of Vagina in Male Perineum with Autologous Tissue Substitute, Open Approach.
No Exclusionary Dx	ICD-10	0W4M0J0	Creation of Vagina in Male Perineum with Synthetic Substitute, Open Approach
No Exclusionary Dx	ICD-10	0W4M0K0	Creation of Vagina in Male Perineum with Non-autologous Tissue Substitute, Open Approach
No Exclusionary Dx	ICD-10	0W4M0Z0	Creation of Vagina in Male Perineum, Open Approach
No Exclusionary Dx	ICD-10	0W4N071	Creation of Penis in Female Perineum with Autologous Tissue Substitute, Open Approach
No Exclusionary Dx	ICD-10	0W4N0J1	Creation of Penis in Female Perineum with Synthetic Substitute, Open Approach
No Exclusionary Dx	ICD-10	0W4N0K1	Creation of Penis in Female Perineum with Non-autologous Tissue Substitute, Open Approach
No Exclusionary Dx	ICD-10	0W4N0Z1	Creation of Penis in Female Perineum, Open Approach
No Exclusionary Dx	CPT	56805	Clitoroplasty for intersex state
No Exclusionary Dx	CPT	57291	Construction of artificial vagina; without graft
No Exclusionary Dx	CPT	57292	Construction of artificial vagina; with graft
No Exclusionary Dx	CPT	57335	Vaginoplasty for intersex state
No Exclusionary Dx	CPT	55175	Scrotoplasty; simple
No Exclusionary Dx	CPT	55180	Scrotoplasty; Complicated
No Exclusionary Dx	CPT	55970	Intersex surgery; male to female
No Exclusionary Dx	CPT	55980	Intersex surgery-Female to Male
Reproductive Organ	CPT	58661	Laparoscopic Procedures on the Oviduct/Ovary
Reproductive Organ	CPT	53430	Urethroplasty (reconstruction of female urethra)
Reproductive Organ	CPT	53420	Urethroplasty, 2-stage reconstruction or repair of prostatic or membranous urethra; first stage
Reproductive Organ	CPT	53425	Urethroplasty, 2-stage reconstruction or repair of prostatic or membranous urethra; second stage
Reproductive Organ	CPT	58152	Total abdominal hysterectomy (corpus and cervix), with or without removal of tube(s), with or without removal of ovary(s);
Reproductive Organ	CPT	58150	Total abdominal hysterectomy (corpus and cervix), with or without removal of tube(s), with or without removal of ovary(s);
Reproductive Organ	CPT	58180	Supracervical abdominal hysterectomy (subtotal hysterectomy), with or without removal of tube(s), with or without removal of ovary(s)
Reproductive Organ	CPT	58260	Vaginal hysterectomy, for uterus 250 g or less;
Reproductive Organ	CPT	58262	Vaginal hysterectomy, for uterus 250 g or less; with removal of tube(s), and/or ovary(s)
Reproductive Organ	CPT	58263	Vaginal hysterectomy, for uterus 250 g or less; with removal of tube(s), and/or ovary(s); and repair of enterocele
Reproductive Organ	CPT	58267	Vaginal hysterectomy, for uterus 250 g or less;
Reproductive Organ	CPT	58270	Vaginal hysterectomy, for uterus 250 g or less; with repair of enterocele
Reproductive Organ	CPT	58275	Vaginal hysterectomy, with total or partial vaginectomy;
Reproductive Organ	CPT	58280	Vaginal hysterectomy, with total or partial vaginectomy; and repair of enterocele
Reproductive Organ	CPT	58290	Vaginal hysterectomy, for uterus greater than 250 g;

Reproductive Organ	CPT	58291	Vaginal hysterectomy, for uterus greater than 250 g; with removal of tube(s) and/or ovary(s)
Reproductive Organ	CPT	58292	Vaginal hysterectomy, for uterus greater than 250 g; with removal of tube(s) and/or ovary(s); and repair of enterocele
Reproductive Organ	CPT	58294	Vaginal hysterectomy, for uterus greater than 250 g; and repair of enterocele
Reproductive Organ	CPT	58541	Laparoscopy, surgical, supracervical hysterectomy, for uterus 250 g or less;
Reproductive Organ	CPT	58542	Laparoscopy, surgical, supracervical hysterectomy, for uterus 250 g or less; with removal of tube(s) and/or ovary(s)
Reproductive Organ	CPT	58543	Laparoscopy, surgical, supracervical hysterectomy, for uterus greater than 250 g;
Reproductive Organ	CPT	58544	Laparoscopy, surgical, supracervical hysterectomy, for uterus greater than 250 g; with removal of tube(s) and/or ovary(s)
Reproductive Organ	CPT	58550	Laparoscopy, surgical, with vaginal hysterectomy, for uterus 250 g or less;
Reproductive Organ	CPT	58552	Laparoscopy, surgical, with vaginal hysterectomy, for uterus 250 g or less; with removal of tube(s) and/or ovary(s)
Reproductive Organ	CPT	58553	Laparoscopy, surgical, with vaginal hysterectomy, for uterus greater than 250 g;
Reproductive Organ	CPT	58554	Laparoscopy, surgical, with vaginal hysterectomy, for uterus greater than 250 g; with removal of tube(s) and/or ovary(s)
Reproductive Organ	CPT	58570	Laparoscopy, surgical, with total hysterectomy, for uterus 250 g or less;
Reproductive Organ	CPT	58571	Laparoscopy, surgical, with total hysterectomy, for uterus 250 g or less; with removal of tube(s) and/or ovary(s)
Reproductive Organ	CPT	58572	Laparoscopy, surgical, with total hysterectomy, for uterus greater than 250 g;
Reproductive Organ	CPT	58573	Laparoscopy, surgical, with total hysterectomy, for uterus greater than 250 g; with removal of tube(s) and/or ovary(s)
Reproductive Organ	CPT	57111	Vaginectomy; with removal of paravaginal tissue (radical vaginectomy)
Reproductive Organ	CPT	57110	Vaginectomy, complete removal of vaginal wall
Reproductive Organ	CPT	57106	Vaginectomy, partial removal of vaginal wall
Reproductive Organ	CPT	56620	Vulvectomy; simple
Reproductive Organ	CPT	56625	Vulvectomy simple; complete
Reproductive Organ	CPT	15750	Neurovascular pedicle flap for metoidioplasty with clitoral transposition; other procedures utilize flaps and or free skin grafts
Reproductive Organ	CPT	56800	Plastic repair of introitus
Reproductive Organ	CPT	56810	Perineoplasty, repair of perineum, non-obstetrical
Reproductive Organ	CPT	58720	Salpingo-oophorectomy, complete or partial, unilateral or bilateral (separate procedure)
Reproductive Organ	CPT	58940	Oophorectomy, complete or partial, unilateral or bilateral (separate procedure)
Reproductive Organ	ICD-10	0UTG0ZZ	Resection of vagina, open approach
Reproductive Organ	ICD-10	0UTG4ZZ	Resection of vagina, percutaneous endoscopic approach
Reproductive Organ	ICD-10	0UTG7ZZ	Resection of vagina, via natural or artificial opening
Reproductive Organ	ICD-10	0UTG8ZZ	Resection of vagina, via natural or artificial opening endoscopic
Reproductive Organ	ICD-10	0UTM0ZZ	Resection of vulva, open approach
Reproductive Organ	ICD-10	0UTMXZZ	Resection of vulva, external approach
Reproductive Organ	CPT	53410	Urethroplasty - reconstruction of male anterior urethra
Reproductive Organ	CPT	54520	Excision procedures on the testis
Reproductive Organ	CPT	54690	Laparoscopic procedures on the testis

© 2024 Dai D, et al. *JAMA Network Open.*

Reproductive Organ	CPT	54660	Testicular implants
Reproductive Organ	CPT	54530	Orchiectomy
Reproductive Organ	CPT	55180	Scrotoplasty
Reproductive Organ	CPT	55175	Scrotoplasty; simple
Reproductive Organ	CPT	54120	Amputation of penis; partial
Reproductive Organ	CPT	54125	Penectomy
Reproductive Organ	CPT	15757	Phalloplasty (via free tissue/muscle/skin grafts)
Reproductive Organ	CPT	15734	Phalloplasty (via free tissue/muscle/skin grafts)
Reproductive Organ	CPT	64856	Neurorrhaphy (commonly medial and lateral antebrachial cutaneous nerve [radial forearm] to ileoinguinal and dorsal clitoral nerves)
Reproductive Organ	CPT	54405	Penile prosthesis (multi-component/hydraulic or semi-rigid)
Reproductive Organ	CPT	54400	Penile prosthesis (multi-component/hydraulic or semi-rigid)
Reproductive Organ	ICD-10	0VBC0ZZ	Excision of Bilateral Testes, Open Approach
Reproductive Organ	ICD-10	0VB50ZZ	Excision of Scrotum, Open Approach
Reproductive Organ	ICD-10	0VB53ZZ	Excision of Scrotum, Percutaneous Approach
Reproductive Organ	ICD-10	0VB54ZZ	Excision of Scrotum, Percutaneous Endoscopic Approach
Reproductive Organ	ICD-10	0VB5XZZ	Excision of Scrotum, External Approach
Reproductive Organ	ICD-10	0VBS0ZZ	Excision of Penis, Open Approach
Reproductive Organ	ICD-10	0VBSXZZ	Excision of Penis, External Approach
Reproductive Organ	ICD-10	0VTS0ZZ	Resection of penis, open approach
Reproductive Organ	ICD-10	0VTS4ZZ	Resection of penis, percutaneous endoscopic approach
Reproductive Organ	ICD-10	0VTSXZZ	Resection of penis, external approach
Chest	CPT	19300	The provider excises the patient's breast to treat excessive enlargement of the male breast (gynecomastia).
Chest	CPT	19303	Mastectomy, simple, complete
Chest	CPT	19304	Subcutaneous mastectomy with nipple preservation and no nipple transposition
Chest	CPT	19318	Breast reduction with pedicle or free nipple graft (in a free nipple graft procedure, a small de-epithelialized flap may be retained to provide contour to the subareola)
Chest	CPT	19357	Tissue expander placement in breast reconstruction, including subsequent expansion(s)
Chest	CPT	19324	Breast augmentation without prosthetic implant, e.g., fat grafting
Chest	CPT	19325	Breast augmentation with prosthetic implant
Chest	CPT	19316	Mastopexy
Chest	CPT	19350	Nipple/areola reconstruction
Head	CPT	31750	Tracheoplasty; cervical
Head	CPT	21125	Augmentation, mandibular body or angle; prosthetic material
Head	CPT	21127	Augmentation, mandibular body or angle; with bone graft, onlay or inter-positional (includes obtaining autograft)
Head	CPT	21208	Osteoplasty, facial bones; augmentation (autograft, allograft, or prosthetic implant)
Head	CPT	21209	Osteoplasty, facial bones; reduction
Head	CPT	30400	Rhinoplasty, primary; lateral and alar cartilages and/or elevation of nasal tip

© 2024 Dai D, et al. *JAMA Network Open.*

Head	CPT	30410	Rhinoplasty, primary; complete, external parts including bony pyramid, lateral and alar cartilages, and/or elevation of nasal tip
Head	CPT	30420	Rhinoplasty, primary; including major septal repair
Head	CPT	21120	Genioplasty; augmentation (autograft, allograft, prosthetic material)
Head	CPT	21121	Genioplasty; sliding osteotomy, single piece
Head	CPT	21122	Genioplasty; sliding osteotomies, 2 or more osteotomies (e.g., wedge excision or bone wedge reversal for asymmetrical chin)
Head	CPT	21123	Genioplasty; sliding, augmentation with inter-positional bone grafts (includes obtaining autografts)
Head	CPT	15820	Blepharoplasty, lower eyelid;
Head	CPT	15821	Blepharoplasty, lower eyelid; with extensive herniated fat pad
Head	CPT	15822	Blepharoplasty, upper eyelid;
Head	CPT	15823	Blepharoplasty, upper eyelid; with excessive skin weighting down lid
Head	CPT	15824	Rhytidectomy; forehead
Head	CPT	15825	Rhytidectomy; neck with platysmal tightening (platysmal flap, p-flap)
Head	CPT	15826	Rhytidectomy; glabellar frown lines
Head	CPT	15828	Rhytidectomy; cheek, chin, and neck
Head	CPT	30430	Rhinoplasty, secondary; minor revision (small amount of nasal tip work)
Head	CPT	30435	Rhinoplasty, secondary; intermediate revision (bony work with osteotomies)
Head	CPT	30450	Rhinoplasty, secondary; major revision (nasal tip work and osteotomies)
Head	CPT	15829	Rhytidectomy; superficial musculoaponeurotic system (smas) flap
Head	CPT	21137	Reduction forehead; contouring only
Head	CPT	21138	Reduction forehead; contouring and application of prosthetic material or bone graft (includes obtaining autograft)
Head	CPT	21139	Reduction forehead; contouring and setback of anterior frontal sinus wall
Head	CPT	67900	Repair of brow ptosis (supraciliary, mid-forehead or coronal approach)
Head	CPT	21270	Malar augmentation, prosthetic material
Head	CPT	31599	Unlisted procedure, larynx

Note: Procedural codes were compiled from gender-affirming surgery lists from Aetna, Blue Cross Blue Shield, and the Centers of Medicare and Medicaid services as well as a prior study. [1-4] Only procedure codes that alone can be considered gender-affirming were included in the list above and used to identify receipt gender-affirming surgery. For example, procedure codes for skin grafts or injection of filling material were not included in the list above.

eTable 2: TGD-related diagnoses

Code	Description
F64	Gender identity disorders
F64.0	Transsexualism
F64.1	Dual role transvestism

© 2024 Dai D, et al. *JAMA Network Open.*

F64.2	Gender identity disorder of childhood
F64.8	Other gender identity disorders
F64.9	Gender identity disorder, unspecified
F65.1	Transvestic fetishism
Z87.890	Personal history of sex reassignment

Note: TGD-related diagnoses were defined from prior work and insurance coverage lists.[1-3,5,6]

© 2024 Dai D, et al. *JAMA Network Open.*

eTable 3: Exclusionary diagnoses for ruling out gender-affirming surgery, by procedure type

Procedure Type	ICD-10 codes starting with	Description
Reproductive Organ	C51	Malignant neoplasm of vulva
Reproductive Organ	C52	Malignant neoplasm of vagina
Reproductive Organ	C56	Malignant neoplasm of the ovary
Reproductive Organ	C570	Malignant neoplasm of fallopian tube
Reproductive Organ	C574	Malignant neoplasm of uterine adnexa, unspecified
Reproductive Organ	C577	Malignant neoplasm of other specified female genital organs
Reproductive Organ	C578	Malignant neoplasm of overlapping sites of female genital organs
Reproductive Organ	C579	Malignant neoplasm of female genital organ, unspecified
Reproductive Organ	C60	Malignant neoplasm of penis
Reproductive Organ	C62	Malignant neoplasm of testis
Reproductive Organ	C63	Malignant neoplasm of other and unspecified male genital organs
Reproductive Organ	D06	Carcinoma in situ of cervix uteri
Reproductive Organ	D176	Benign lipomatous neoplasm of spermatic cord
Reproductive Organ	D25	Leiomyoma of uterus
Reproductive Organ	D26	Other benign neoplasms of uterus
Reproductive Organ	D27	Benign neoplasm of ovary
Reproductive Organ	D28	Benign neoplasm of other and unspecified female genital organs
Reproductive Organ	D29	Benign neoplasm of male genital organs
Reproductive Organ	D30	Benign neoplasm of urinary organs
Reproductive Organ	D3616	Benign neoplasm of peripheral nerves and autonomic nervous system of pelvi
Reproductive Organ	D39	Neoplasm of uncertain or unknown behaviour of female genital organs
Reproductive Organ	D495	Neoplasm of unspecified behavior of other genitourinary organs
Reproductive Organ	E28	Ovarian dysfunction
Reproductive Organ	N27	Inflammatory disease of cervix uteri
Reproductive Organ	N35	Urethral stricture
Reproductive Organ	N36	Other disorders of urethra
Reproductive Organ	N40	Benign prostatic hyperplasia
Reproductive Organ	N41	Inflammatory disease of prostate
Reproductive Organ	N42	Other and unspecified disorders of prostate
Reproductive Organ	N43	Hydrocele and spermatocele
Reproductive Organ	N44	Noninflammatory disorders of testis
Reproductive Organ	N45	Orchitis and epididymitis
Reproductive Organ	N47	Disorders of prepuce
Reproductive Organ	N48	Other disorders of penis
Reproductive Organ	N49	Inflammatory disorders of male genital organs, not elsewhere classified

© 2024 Dai D, et al. *JAMA Network Open.*

Reproductive Organ	N501	Hematocele
Reproductive Organ	N503	Cyst of epididymis
Reproductive Organ	N70	Salpingitis and oophoritis
Reproductive Organ	N71	Inflammatory disease of uterus, except cervix
Reproductive Organ	N72	Inflammatory disease of cervix uteri
Reproductive Organ	N73	Other female pelvic inflammatory diseases
Reproductive Organ	N74	Female pelvic inflammatory disorders in diseases classified elsewhere
Reproductive Organ	N75	Diseases of Bartholin's gland
Reproductive Organ	N76	Other inflammation of vagina and vulva
Reproductive Organ	N77	Vulvovaginal ulceration and inflammation in diseases classified elsewhere
Reproductive Organ	N81	Female genital prolapse
Reproductive Organ	N82	Fistulae involving female genital tract
Reproductive Organ	N83	Noninflammatory disorders of ovary, fallopian tube and broad ligament
Reproductive Organ	N84	Polyp of female genital tract
Reproductive Organ	N85	Other noninflammatory disorders of uterus, except cervix
Reproductive Organ	N87	Dysplasia of cervix uteri
Reproductive Organ	N88	Other noninflammatory disorders of cervix uteri
Reproductive Organ	N89	Other noninflammatory disorders of vagina
Reproductive Organ	N90	Other noninflammatory disorders of vulva and perineum
Reproductive Organ	Q50	Congenital malformations of ovaries, fallopian tubes and broad ligaments
Reproductive Organ	Q51	Congenital malformations of uterus and cervix
Reproductive Organ	Q52	Other congenital malformations of female genitalia
Reproductive Organ	Q53	Undescended and ectopic testicle
Reproductive Organ	Q54	Hypospadias
Reproductive Organ	Q55	Other congenital malformations of male genital organs
Reproductive Organ	Q64	Other congenital malformations of urinary system
Reproductive Organ	R1900	Intra-abdominal and pelvic swelling, mass and lump
Reproductive Organ	S3140XA	Unspecified open wound of vagina and vulva, initial encounter
Reproductive Organ	S31501A	Unspecified open wound of unspecified external genital organs, male, initial encounter
Reproductive Organ	S358X1S	Laceration of other blood vessels at abdomen, lower back and pelvis level, initial encounter
Reproductive Organ	S3713XA	Laceration of ureter, initial encounter
Reproductive Organ	S3739XA	Other injury of urethra, initial encounter
Reproductive Organ	S3769XA	Other injury of uterus, initial encounter
Reproductive Organ	Z1502	Genetic susceptibility to malignant neoplasm of ovary
Reproductive Organ	Z1504	Genetic susceptibility to malignant neoplasm of endometrium
Reproductive Organ	Z4002	Encounter for prophylactic removal of ovary
Reproductive Organ	Z4003	Encounter for prophylactic removal of fallopian tube
Reproductive Organ	Z804	Family history of malignant neoplasm of genital organs

© 2024 Dai D, et al. *JAMA Network Open.*

Reproductive Organ	Z805	Family history of malignant neoplasm of urinary tract
Reproductive Organ	Z854	Personal history of malignant neoplasm of genital organs
Reproductive Organ	Z855	Personal history of malignant neoplasm of urinary tract
Reproductive Organ	Z86001	Personal history of in-situ neoplasm of cervix uteri
Reproductive Organ	Z86002	Personal history of in-situ neoplasm of other and unspecified genital organs
Reproductive Organ	Z874	Personal history of other diseases of the genitourinary system
Chest	N6482<br>NOTE: Only exclude patients with this diagnosis if they underwent augmentation or reduction, not mastectomy.	Hypoplasia
Chest	C50	Malignant neoplasm of breast
Chest	C761	Malignant neoplasm of thorax
Chest	C7951	Secondary malignant neoplasm of bone
Chest	D05	Carcinoma in situ of breast
Chest	D24	Benign neoplasm of breast
Chest	D493	Neoplasm of unspecified behavior of breast
Chest	N60	Benign mammary dysplasia
Chest	N61	Inflammatory disorders of breast
Chest	N63	Unspecified lump in breast
Chest	N640	Fissure and fistula of nipple
Chest	N641	Fat necrosis of breast
Chest	N643	Galactorrhea not associated with childbirth
Chest	N6451	Induration of breast
Chest	Q83	Congenital malformation of breast
Chest	R920	Mammographic microcalcification found on diagnostic imaging of breast
Chest	S2100	Unspecified open wound of breast
Chest	Z421	Encounter for breast reconstruction following mastectomy
Chest	Z803	Family history of malignant neoplasm of breast
Chest	Z853	Personal history of malignant neoplasm of breast
Chest	Z86000	Personal history of in-situ neoplasm of breast
Head	C08	Malignant neoplasm of salivary ducts
Head	C760	Malignant neoplasm of head, face and neck
Head	D106	Benign neoplasm of nasopharynx
Head	D210	Benign neoplasm of connective and other soft tissue of head, face and neck
Head	G4730	Sleep apnea, unspecified
Head	G4733	Obstructive sleep apnea

© 2024 Dai D, et al. *JAMA Network Open.*

Head	J33	Nasal polyp
Head	J342	Deviated nasal septum
Head	J343	Hypertrophy of nasal turbinates
Head	J341	Cyst and mucocele of nose and nasal sinus
Head	J3481	Nasal mucositis (ulcerative)
Head	J352	Hypertrophy of adenoids
Head	M26609	Temporomandibular joint disorders
Head	M950	Acquired deformity of nose
Head	Q315	Congenital laryngomalacia
Head	Q321	Congenital malformations of trachea
Head	Z8521	Personal history of malignant neoplasm of larynx
Head	Z8522	Personal history of malignant neoplasm of nasal cavities, middle ear, and accessory sinuses
Head	Z8581	Personal history of malignant neoplasm of lip, oral cavity, and pharynx
Head	Z85850	Personal history of malignant neoplasm of thyroid

eTable 4: Diagnoses indicating manually review of patient history is necessary to determine whether a surgery was definitively gender-affirming

Procedure Type	ICD-10 codes starting with	Description
Chest	N6459	Other signs and symptoms in breast
Chest	N642	Atrophy of breast
Reproductive Organ	N508	Other specified disorders of male genital organs

eTable 5: Intersex exclusionary diagnoses that rule out gender-affirming surgery

Procedure Type	ICD-10 Codes starting with	Description
Intersex	Q991	True hermaphrodite
Intersex	E25	Congenital adrenogenital disorders associated with enzyme deficiency
Intersex	Q96-Q99	Pseudohermaphorditism w specified chromosomal anomaly
Intersex	E291	Testicular hypofunction
Intersex	Q891	Congenital malformations of adrenal gland
Intersex	Q990	Chimera 46, XX/46, XY
Intersex	Q526	Congenital malformation of clitoris
Intersex	Q51818	Other congenital malformations of uterus
Intersex	Q510	Agenesis and aplasia of uterus

Intersex	Q878	Mayer-Rokitansky-Küster-Hauser syndrome type 2<br>Disease definition
Intersex	E345	Androgen insensitivity syndrome
Intersex	Q532	Undescended testicle, bilateral
Intersex	Q542	Hypospadias, penoscrotal
Intersex	Q543	Hypospadias, perineal
Intersex	Q5500	Absence and aplasia of testis
Intersex	Q5501	Anorchia
Intersex	Q561	Male pseudohermaphroditism, not elsewhere classified
Intersex	Q973	Female with 46,XY karyotype
Intersex	Q520	Congenital absence of vagina
Intersex	Q521	Doubling of vagina
Intersex	Q562	Female pseudohermaphroditism, not elsewhere classified
Intersex	Q983	Other male with 46,XX karyotype
Intersex	E2500	Salt-losing congenital adrenal hyperplasia
Intersex	E2501	Congenital adrenal hyperplasia
Intersex	Q960-Q969	Turner syndrome and variants
Intersex	Q970-Q979	Other sex chromosome abnormalities, female phenotype, not elsewhere classified
Intersex	Q980,Q981,Q982,Q984	Klinefelter syndrome and variants
Intersex	Q985-Q989	Other sex chromosome abnormalities, male phenotype, not elsewhere classified
Intersex	E259	Adrenogenital disorder, unspecified
Intersex	Q560	Hermaphroditism, not elsewhere classified
Intersex	Q563	Pseudohermaphroditism, unspecified
Intersex	Q564	Indeterminate sex, unspecified
Intersex	Q530	Ectopic testis
Intersex	Q531	Undescended testicle, unilateral
Intersex	Q539	Undescended testicle, unspecified
Intersex	Q540	Hypospadias, balanic
Intersex	Q541	Hypospadias, penile
Intersex	Q544	Congenital chordee
Intersex	Q548	Other hypospadias
Intersex	Q549	Hypospadias, unspecified
Intersex	Q551	Hypoplasia of testis and scrotum
Intersex	Q5520	Retractile testis
Intersex	Q5528	Unspecified congenital malformations of testis and scrotum
Intersex	Q556	Other congenital malformations of penis
Intersex	Q558	Other specified congenital malformations of male genital organs
Intersex	Q559	Congenital malformation of male genital organ, unspecified
Intersex	Q522	Congenital rectovaginal fistula

© 2024 Dai D, et al. *JAMA Network Open.*

Intersex	Q523	Imperforate hymen
Intersex	Q524	Other congenital malformations of vagina
Intersex	Q525	Fusion of labia
Intersex	Q528	Other specified congenital malformations of female genitalia
Intersex	Q529	Congenital malformation of female genitalia, unspecified
Intersex	Q641	Exstrophy of urinary bladder
Intersex	Q437	Persistent cloaca
Intersex	Q830	Congenital absence of breast with absent nipple

Note: The diagnosis codes used to identify intersex people draw from previous work.[10,11]

© 2024 Dai D, et al. *JAMA Network Open.*

References

1. Hughto JMW, Hughes L, Yee K, et al. Improving Data-Driven Methods to Identify and Categorize Transgender Individuals by Gender in Insurance Claims Data. *LGBT Health*. May-Jun 2022;9(4):254-263. doi:10.1089/lgbt.2021.0433
2. Centers for Medicare & Medicaid Services. Billing and Coding: Gender Reassignment Services for Gender Dysphoria. Centers for Medicare & Medicaid Services,. Accessed June 29, 2023, 2023. https://www.cms.gov/medicare-coverage-database/view/article.aspx?articleid=53793&ver=25&=
3. Blue Cross Blue Shield of Massachusetts. *Gender Affirming Services (Transgender and Gender Diverse Services)*. Vol. 189. 2023. https://www.bluecrossma.org/medical-policies/sites/g/files/csphws2091/files/acquiadam-assets/189%20Gender%20Affirming%20Services%20%28Transgender%20Services%29%20prn.pdf
4. Aetna. Gender Affirming Surgery. Aetna. Updated August 8, 2023. Accessed June 1, 2023, 2023. https://www.aetna.com/cpb/medical/data/600_699/0615.html#dummyLink1
5. Blosnich JR, Cashy J, Gordon AJ, et al. Using clinician text notes in electronic medical record data to validate transgender-related diagnosis codes. *J Am Med Inform Assoc*. Jul 1 2018;25(7):905-908. doi:10.1093/jamia/ocy022
6. Ewald ER, Guerino P, Dragon C, Laffan AM, Goldstein Z, Streed C, Jr. Identifying Medicare Beneficiaries Accessing Transgender-Related Care in the Era of ICD-10. *LGBT Health*. May/Jun 2019;6(4):166-173. doi:10.1089/lgbt.2018.0175
7. Coleman E, Radix AE, Bouman WP, et al. Standards of care for the health of transgender and gender diverse people, Version 8. *Int J Transgend Health*. 2022;23(Suppl 1):S1-s259. doi:10.1080/26895269.2022.2100644
8. Tracking the rise of anti-trans bills in the U.S. Trans Legislation Tracker. June 14, 2023, Accessed June 14, 2023, 2023. https://translegislation.com/learn
9. Carpenter M. The human rights of intersex people: addressing harmful practices and rhetoric of change. *Reprod Health Matters*. May 2016;24(47):74-84. doi:10.1016/j.rhm.2016.06.003
10. Kohva E, Miettinen PJ, Taskinen S, Hero M, Tarkkanen A, Raivio T. Disorders of sex development: timing of diagnosis and management in a single large tertiary center. *Endocr Connect*. Apr 2018;7(4):595-603. doi:10.1530/ec-18-0070
11. Global Action for Trans Equality. *Submission by GATE to the World Health Organization: Intersex codes in the International Classification of Diseases (ICD) 11 Beta Draft*. 2017. June, 2017. Accessed June 1, 2023. https://morgancarpenter.com/consulting/

© 2024 Dai D, et al. *JAMA Network Open.*
