# Supplementary material for: Prevalence of Gender-Affirming Surgical Procedures Among Minors and Adults in the US
Source: JAMA Netw Open. 2024 Jun 27;7(6):e2418814. doi: 10.1001/jamanetworkopen.2024.18814 (PMC11211955; doi:10.1001/jamanetworkopen.2024.18814)
Supplement: Supplement 2. — Data Sharing Statement [file jamanetwopen-e2418814-s002.pdf]

Data Sharing Statement

Data

Data available: No

Additional Information

Explanation for why data not available: Our data use agreement would not allow for this.
